# Tools used to assess the quality of peer review reports: a methodological systematic review

**DOI:** 10.1186/s12874-019-0688-x

**Published:** 2019-03-06

**Authors:** Cecilia Superchi, José Antonio González, Ivan Solà, Erik Cobo, Darko Hren, Isabelle Boutron

**Affiliations:** 1 Department of Statistics and Operations Research, Barcelona-Tech, UPC, c/ Jordi Girona 1-3, 08034 Barcelona, Spain; 2 INSERM, U1153 Epidemiology and Biostatistics Sorbonne Paris Cité Research Center (CRESS), Methods of therapeutic evaluation of chronic diseases Team (METHODS), F-75014 Paris, France; 3 Paris Descartes University, Sorbonne Paris Cité, Paris, France; 4 Iberoamerican Cochrane Centre, Hospital de la Santa Creu i Sant Pau, C/ Sant Antoni Maria Claret 167, Pavelló 18 - planta 0, 08025 Barcelona, Spain; 5 CIBER de Epidemiología y Salud Pública (CIBERESP), Madrid, Spain; 6 Department of Psychology, Faculty of Humanities and Social Sciences, University of Split, Split, Croatia; 7 Centre d’épidémiologie Clinique, Hôpital Hôtel-Dieu, 1 place du Paris Notre-Dame, 75004 Paris, France

**Keywords:** Peer review, Quality control, Methods, Report, Systematic review

## Abstract

**Background:**

A strong need exists for a validated tool that clearly defines peer review report quality in biomedical research, as it will allow evaluating interventions aimed at improving the peer review process in well-performed trials. We aim to identify and describe existing tools for assessing the quality of peer review reports in biomedical research.

**Methods:**

We conducted a methodological systematic review by searching PubMed, EMBASE (via Ovid) and The Cochrane Methodology Register (via The Cochrane Library) as well as Google® for all reports in English describing a tool for assessing the quality of a peer review report in biomedical research. Data extraction was performed in duplicate using a standardized data extraction form. We extracted information on the structure, development and validation of each tool. We also identified quality components across tools using a systematic multi-step approach and we investigated quality domain similarities among tools by performing hierarchical, complete-linkage clustering analysis.

**Results:**

We identified a total number of 24 tools: 23 scales and 1 checklist. Six tools consisted of a single item and 18 had several items ranging from 4 to 26. None of the tools reported a definition of ‘quality’. Only 1 tool described the scale development and 10 provided measures of validity and reliability. Five tools were used as an outcome in a randomized controlled trial (RCT). Moreover, we classified the quality components of the 18 tools with more than one item into 9 main quality domains and 11 subdomains. The tools contained from two to seven quality domains. Some domains and subdomains were considered in most tools such as the *detailed/thorough* (11/18) nature of reviewer’s comments. Others were rarely considered, such as whether or not the reviewer made comments on the *statistical methods* (1/18).

**Conclusion:**

Several tools are available to assess the quality of peer review reports; however, the development and validation process is questionable and the concepts evaluated by these tools vary widely. The results from this study and from further investigations will inform the development of a new tool for assessing the quality of peer review reports in biomedical research.

**Electronic supplementary material:**

The online version of this article (10.1186/s12874-019-0688-x) contains supplementary material, which is available to authorized users.

## Background

The use of editorial peer review originates in the eighteenth century [1]. It is a longstanding and established process that generally aims to provide a fair decision-making mechanism and improve the quality of a submitted manuscript [2]. Despite the long history and application of the peer review system, its efficacy is still a matter of controversy [3–7]. About 30 years after the first international Peer Review Congress, there are still ‘scarcely any bars to eventual publication. There seems to be no study too fragmented, no hypothesis too trivial [...] for a paper to end up in print’ (Drummond Rennie, chair of the advisory board) [8].

Recent evidence suggests that many current editors and peer reviewers in biomedical journals still lack the appropriate competencies [9]. In particular, it has been shown that peer reviewers rarely receive formal training [3]. Moreover, their capacity to detect errors [10, 11], identify deficiencies in reporting [12] and spin [13] has been found lacking.

Some systematic reviews have been performed to estimate the effect of interventions aimed at improving the peer review process [2, 14, 15]. These studies showed that there is still a lack of evidence supporting the use of interventions to improve the quality of the peer review process. Furthermore, Bruce and colleagues highlighted the urgent need to clarify outcomes, such as peer review report quality, that should be used in randomized controlled trials evaluating these interventions [15].

A validated tool that clearly defines peer review report quality in biomedical research is greatly needed. This will allow researchers to have a structured instrument to evaluate the impact of interventions aimed at improving the peer review process in well-performed trials. Such a tool could also be regularly used by editors to evaluate the work of reviewers.

Herein, as starting point for the development of a new tool, we identify and describe existing tools that assess the quality of peer review reports in biomedical research.

## Methods

### Study design

We conducted a methodological systematic review and followed the standard Preferred Reporting Items for Systematic Review and Meta-Analysis (PRISMA) guidelines [16]. The quality of peer review reports is an outcome that in the long term is related to clinical relevance and patient care. However, the protocol was not registered in PROSPERO, as this review does not contain direct health-related outcomes [17].

### Information sources and search strategy

We searched PubMed, EMBASE (via Ovid) and The Cochrane Methodology Register (via The Cochrane Library) from their inception to October 27, 2017 as well as Google® (search date: October 20, 2017) for all reports describing a tool to assess the quality of a peer review report in biomedical research. Search strategies were refined in collaboration with an expert methodologist (IS) and are presented in the Additional file 1. We hand-searched the citation lists of included papers and consulted a senior editor with expertise in editorial policies and peer review processes to further identify relevant reports.

### Eligibility criteria

We included all reports describing a tool to assess the quality of a peer review report. Sanderson and colleagues defined a tool as ‘any structured instrument aimed at aiding the user to assess the quality [...]’ [18]. Building on this definition, we defined a quality tool as any structured or unstructured instrument assisting the user to assess the quality of peer review report (for definitions see Table 1). We restricted inclusion to the English language.

**Table 1:** Definition of terms used in the present study

Structured quality tool: scale or checklist including more than one item aimed at guiding the user to assess the overall quality of a peer review report.	
Unstructured quality tool: scale or checklist including only one item inquiring the overall quality of a peer review report.	
Items: elements of a scale or checklist representing a component of peer review report quality. Items in a scale could or could not have an attached numerical score. If there is no attached score, these items provide the evaluator with a guidance to assess the overall quality of a peer review report.	
Overall quality score in a scale is measured as: • Sum of scores: score obtained by summing all scores for each item present in a scale. • Mean of scores: score obtained by dividing the sum of scores for each item with the total number of items included in the tool. • Single score: score obtained in those scales based on a single item. • Summary score: score obtained in those scales with more than one item deriving from a question inquiring the overall quality of peer review report.	

### Study selection

We exported the references retrieved from the search into the reference manager Endnote X7 (Clarivate Analytics, Philadelphia, United States), which was subsequently used to remove duplicates. We reviewed all records manually to verify and remove duplicates that had not been previously detected. A reviewer (CS) screened all titles and abstracts of the retrieved citations. A second reviewer (JAG) carried out quality control on a 25% random sample obtained using the statistical software R 3.3.3 [19]. We obtained and independently examined the full-text copies of potentially eligible reports for further assessment. In the case of disagreement, consensus was determined by a discussion or by involving a third reviewer (DH). We reported the result of this process through a PRISMA flowchart [16]. When several tools were reported in the same article, they were included as separate tools. When a tool was reported in more than one article, we extracted data from all related reports.

### Data extraction

#### General characteristics of tools

We designed a data extraction form using Google® Docs and extracted the general characteristics of the tools. We determined whether the tool was scale or checklist. We defined a tool as a scale when it included a numeric or nominal overall quality score while we considered it as a checklist when an overall quality score was not present. We recorded the total number of items (for definitions see Table 1). For scales with more than 1 item we extracted how items were weighted, how the overall score was calculated, and the scoring range. Moreover, we checked whether the scoring instructions were adequately defined, partially defined, or not defined according to the subjective judgement of two reviewers (CS and JAG) (an example of the definition for scoring instructions is shown in Table 2). Finally, we extracted all information related to the development, validation, and assessment of the tool’s reliability and if the concept of quality was defined.

**Table 2:** Examples of definition of scoring system instructions

Scoring system instructions		
Defined	Partially defined	Not defined
5 (Exceptional) = The rare outstanding critique that is comprehensive, objective, and insightful. Evaluates purpose of the study, study design, scientific validity, and conclusions by numbering questions and constructive suggestions to be addressed by the author. Includes comments to the editor about whether this is something new and important and useful to our readers. 4 (Very good) = Excellent review indicating that the paper was carefully evaluated. Helpful comments to the author and editor with well-documented reasons for decision. 3 (Good) = Useful type of very satisfactory review. Analysis not as well organized, documented, or as complete as above but is reasonable, with adequate comments for the authors. 2 (Below average) = Very brief, superficial evaluation. Reasons for the decision not explained and comments to authors not helpful. 1 (Unacceptable) = Such a poor review that consideration should be given to not sending further papers to this reviewer. Reasons could include evidence of bias, unfair, faulty reasoning, or evaluation (totally disagrees with the opinion of other reviewers and editor) and comments to author either absent, inappropriate, or inadequate to explain how the paper was rated. (Landkroon 2006) [42]	1 (Poor) = Does not follow reviewer guideline structure or preferred formatting in providing comments; unfavourable timeliness. 2 (Acceptable) = Comments are somewhat helpful; review meets timeline. 3 (Reliable) = Thorough and helpful comments; timely submission. 4 (Excellent) = Very strong and detailed comments; review was submitted early or on time; comments enhance the manuscript’s merit and relevance in the field. (Rajesh 2013) [32]	1 = poor; 2 = fair; 3 = good; 4 = excellent (Friedam1995) [22]

Two reviewers (CS and JAG) piloted and refined the data extraction form on a random 5% sample of extracted articles. Full data extraction was conducted by two reviewers (CS and JAG) working independently for all included articles. In the case of disagreement, consensus was obtained by discussion or by involving a third reviewer (DH). Authors of the reports were contacted in cases where we needed further clarification of the tool.

#### Quality components of the peer review report considered in the tools

We followed the systematic multi-step approach recently described by Gentles [20], which is based on a constant comparative method of analysis developed within the Grounded Theory approach [21]. Initially, a researcher (CS) extracted all items included in the tools and for each item identified a ‘key concept’ representing a quality component of peer review reports. Next, two researchers (CS and DH) organized the key concepts into a domain-specific matrix (analogous to the topic-specific matrices described by Gentles). Initially, the matrix consisted of domains for peer review report quality, followed by items representative of each domain and references to literature sources that items were extracted from. As the analysis progressed, subdomains were created and the final version of the matrix included domains, subdomains, items and references.

Furthermore, we calculated the proportions of domains based on the number of items included in each domain for each tool. According to the proportions obtained, we created a domain profile for each tool. Then, we calculated the matrix of Euclidean distances between the domain profiles. These distances were used to perform the hierarchical, complete-linkage clustering analysis, which provided us with a tree structure that we represent in a chart. Through this graphical summary, we were able to identify domain similarities among the different tools, which helped us draw our analytical conclusions. The calculations and graphical representations were obtained using the statistical software R 3.3.3 [19].

## Results

### Study selection and general characteristics of reports

**Fig. 1:**
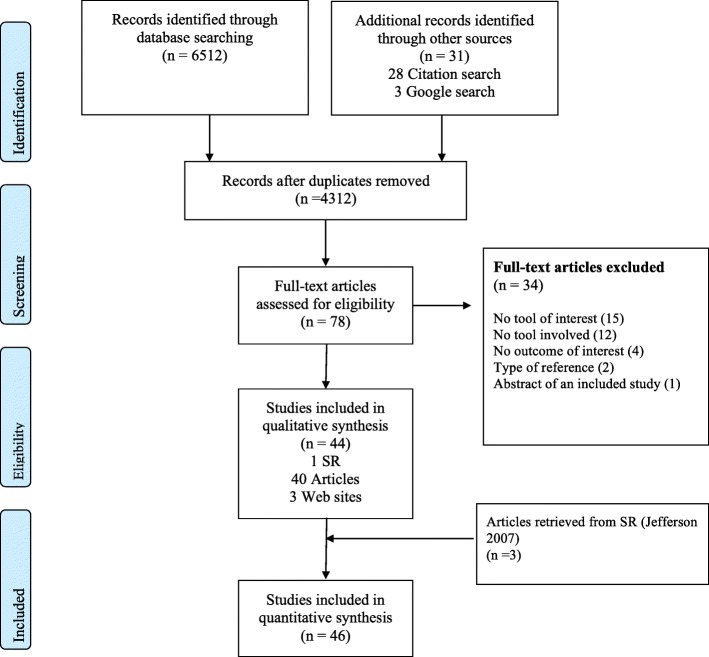
Study selection flow diagram

The screening process is summarized in a flow diagram (Fig.1). Of the 4312 records retrieved, we finally included 46 reports: 39 research articles; 3 editorials; 2 information guides; 1 was a letter to the editor and 1 study was available only as an abstract (excluded studies are listed in Additional file 2; included studies are listed in Additional file 3).

### General characteristics of the tools

In the 46 reports, we identified 24 tools, including 23 scales and 1 checklist. The tools were developed from 1985 to 2017. Four tools had from 2 to 4 versions [22–25]. Five tools were used as an outcome in a randomized controlled trial [23, 25–28]. Table 3 lists the general characteristics of the identified tools. Table 4 presents a more complete descriptive summary of the tools’ characteristics, including types and measures of validity and reliability.

**Table 3:** Main characteristics of the included tools

Characteristics of tools	N (%)
Type of tool:	
Scale	23 (96%)
Checklist	1 (4%)
Number of items:	
1	6 (25%)
> 1	18 (75%)
Weight of items ^a^:	
Same weight	10 (42%)
Different weight	2 (8%)
User defined weight	1 (4%)
Not applicable	11 (46%)^a^
Score System Instruction:	
Defined	5 (21%)
Partially defined	3 (12%)
Not defined	16 (67%)
Tool development:	
Reported	1 (4%)
Not reported	23 (96%)
Overall quality assessment ^b^	
Single score	6 (22%)
Summary score	11 (41%)
Mean score	6 (22%)
Sum score	3 (11%)
Not reported	1 (4%)

^a^Item weight is not applicable for scale with a single item (*n* = 6), checklist (*n* = 1) and for scale including more than one item without a numerical score attached but presenting only a summary score (*n* = 4)

^b^The total number is different because three tools presented more than one way to assess the overall quality and the checklist did not provide an overall score

**Table 4:** Descriptive characteristics of tools used to assess the quality of a peer review report

Journal or Company Name ^a^	First Author, Year	Format	Quality defined ^b^	Overall quality assessment	Items (n)	Items weights ^c^	Scoring range ^d^	Scoring system instruction ^e^	Scale/ Checklist Development ^f^	Validity ^g^	Reliability ^h^	Internal consistency	RCTs ^i^
Advances in Nursing Science; Issues in Mental Health Nursing; The Journal of Holistic Nursing	Shattell 2010 [33]	Scale	N	Summary Score	6	S	1–10	N	NR	NR	NR	NR	0
American Journal of Roentgenology	Friedman 1995 [22]	Scale	N	Single Score	1	NA	1–4	N	NR	NR	NR	NR	0
American Journal of Roentgenology	Kliewer 2005 [49]	Scale	N	Summary Score	4	NA	1–4	N	NR	NR	NR	NR	0
American Journal of Roentgenology	Rajesh 2013 [32]	Scale	N	Single Score	1	NA	1–4	P	NR	NR	NR	NR	0
American Journal of Roentgenology	Berquist 2017 [50]	Scale	N	Summary Score	4	NA	0–4	Y	NR	NR	NR	NR	0
Annals of Emergency Medicine	Callaham 1998 [25]	Scale	N	Single Score	1	NA	1–5	N	NR	NR	Inter-Rater (ICC = 0.44, 0.24, 0.12) ^l^	NR	2 ^m^
Annals of Emergency Medicine	Callaham 2002 [26, 51]	Scale	N	Summary Score	6	NA	1–5	N	NR	NR	Inter-Rater (ICC = 0.44, 0.24, 0.12) ^l^	NR	1
Annals of Emergency Medicine; Annals of Internal Medicine; JAMA; Obstetrics & Gynecology and Ophthalmology	Justice 1998 [35]	Scale	N	Summary Score	4	S	1–5	N	NR	NR	NR	NR	0
British Journal of General Practice	Moore 2014 [29]	Scale	N	Single Score	1	NA	A-E	Y	NR	NR	NR		0
British Medical Journal	Black 1998 (RQI 3.2) [23, 39]	Scale	N	Summary Score	7	S	1–5	N	Y	Face (*N* = 20)	Test-Retest (Kw = 1.00)	Internal Consistency (Cronbach’s alpha = 0.84)	5
				Mean						Content (*N* = 20) Construct	Inter-Rater (Kw = 0.83)		
British Medical Journal	Van Rooyen 1999 (RQI 4) [27]	Scale	N	Mean ^n^	8	S	1–5	N	NR	NR	Inter-Rater (Kw = 0.38–0.67) ^o^		2
Chinese Journal of Tuberculosis and Respiratory Diseases	Yang 2009 [52]	Checklist	N	NA	5	NA	NA	N	NR	NR	NR		0
Journal of Clinical Investigation	Stossel 1985 [30]	Scale	N	Single Score	1	NA	Good- Fair- Poor	Y	NR	NR	NR		0
Journal of General Internal Medicine	McNutt 1990 [28, 40]	Scale	N	Summary Score	9	S	1–5	N	NR	Construct	NR		1
Journal of Vascular Interventional Radiology	Feurer 1994 [41]	Scale	N	Sum	7	D	0–14	N	NR	Content (*N* = 2) Preliminary Criterion (*N* = 2) (Kendall = 0.94)	Inter-Rater (ICC = 0.84)		0
NA	Review quality collector (RQC) 2012 [53]	Scale	N	Mean	4	User-defined weights	0–100	N	NR	NR	NR		0
Nursing Research	Henly 2009 [24]	Scale	N	Mean (CAS, GAS scale)	15	S	1–5	P	NR	NR	Inter-Rater (ICC = 0.79) ^p^		0
				Summary Score (OAS scale)			1–5						
				Summary Score (GRQ scale)			0–100						
Nursing Research	Henly 2010 [36]	Scale	N	Mean (CAS, GAR, SARNR scale)	26	S	1–5	P	NR	NR	Inter-Rater (ICC = 0.75)^p^		0
				Summary Score (GRQ scale)			0–100						
Obstetrics & Gynecology, Dutch Journal of Medicine	Landkroon 2006 [42]	Scale	N	Summary Score	5	NA	1–5	Y	NR	NR	Test-Retest (ICC =0.66–0.88) Inter-Rater (ICC = 0.62)		0
Pakistan Journal of Medical Sciences	Jawaid 2006 [34]	Scale	N	NR ^q^	5	S	1–5	N	NR	NR	NR		0
Peerage of science	Peerage Essay Quality (PEQ) 2011 [37]	Scale	N	Mean	3	S	1–5	N	NR	NR	NR		0
Publons Academy	Review Rating and Feedback Form 2016 [38]	Scale	N	Sum	4	S	0–3 (Full score: 0–12)	N	NR	NR	NR		0
The Journal of Bone and Joint Surgery	Thompson 2016 [31]	Scale	N	Single Score	1	NA	80–100	Y	NR	NR	Inter-Rater (ICC = -4.5 to 0.99) ^r^		0
The National Medical Journal of India	Das Sinha 1999 [54]	Scale	N	Sum	5	D	0–100	N	NR	NR	NR		0

^a^Name of journal or company/organization where the tool was used to assess the quality of their peer review reports

^b^The quality of a peer review report is not clearly defined in any reports

^c^*NA* Not applicable, *S* Same weight for each item, *D* Different weight for each item

^d^*NA* Not applicable

^e^*Y* Yes defined, *P* Partially defined, *N* Not defined

^f, g, h^*NR* Not reported

^i^Number of randomized controlled trials where the tool was used as outcome criteria

^l^The ICC was 0.44 for reviewers, 0.24 for editors, and 0.12 for manuscripts

^m^One article consists of two studies. First study is not a RCT while the second one is a RCT [55]

^n^The overall quality is based on the mean of the first seven items (the item about the tone of the review was not included)

^o^The inter-rater reliability was measured with weighted K for item from 1 to 7 for two editors’ independent assessments

^p^The tool includes more than one scale. We reported inter-rater reliability only for General Review Quality (GRQ) scale

^q^Not reported. Although the authors reported that the reviewers were rated as excellent, good and average based on the quality of the reviews, it is not reported how they assessed the overall quality of peer review reports

^r^ICC range for 11 manuscripts. There was one outlier manuscript that if removed brought the range to 0.87–0.99

Six scales consisted of a single item enquiring into the overall quality of the peer review report, all of them based on directly asking users to score the overall quality [22, 25, 29–32]. These tools assessed the quality of a peer review report by using: 1) a 4 or 5 Likert point scale (*n* = 4); 2) as ‘good’, ‘fair’ and ‘poor’ (*n* = 1); and 3) a restricted scale from 80 to 100 (n = 1). Seventeen scales and one checklist had several items ranging in number from 4 to 26. Of these, 10 used the same weight for each item [23, 24, 27, 28, 33–38]. The overall quality score was the sum of the score for each item (*n* = 3); the mean of the score of the items (*n* = 6); or the summary score (*n* = 11) (for definitions see Table 1). Three scales reported more than one way to assess the overall quality [23, 24, 36]. The scoring system instructions were not defined in 67% of the tools.

None of the tools reported the definition of peer review report quality, and only one described the tool development [39]. The first version of this tool was designed by a development group composed of four researchers and three editors. It was based on a tool used in an earlier study and that had been developed by reviewing the literature and interviewing editors. Successively, the tool was modified by rewording some questions after some group discussions and a guideline for using the tool was drawn up.

Only 3 tools assessed and reported a validation process [39–41]. The assessed types of validity included face validity, content validity, construct validity, and preliminary criterion validity. Face and content validity could involve either a sole editor and author or a group of researchers and editors. Construct validity was assessed with multiple regression analysis using discriminant criteria (reviewer characteristics such as age, sex, and country of residence) and convergent criteria (training in epidemiology and/or statistics); or the overall assessment of the peer review report by authors and an assessment of (*n* = 4–8) specific components of the peer review report by editors or authors. Preliminary criterion was assessed by comparing grades obtained by an editor to those obtained by an editor-in-chief using an earlier version of the tool. Reliability was assessed in 9 tools [24–27, 31, 36, 39, 41, 42]; all reported inter-rater reliability and 2 also reported test-retest reliability. One tool reported the internal consistency measured with the Cronbach’s alpha [39].

### Quality components of the peer review reports considered in the tools with more than one item

We extracted 132 items included in the 18 tools. One item asking for the percentage of co-reviews the reviewer had graded was not included in the classification because it represented a method of measuring reviewer’s performance and not a component of peer review report quality.

**Fig. 2:**
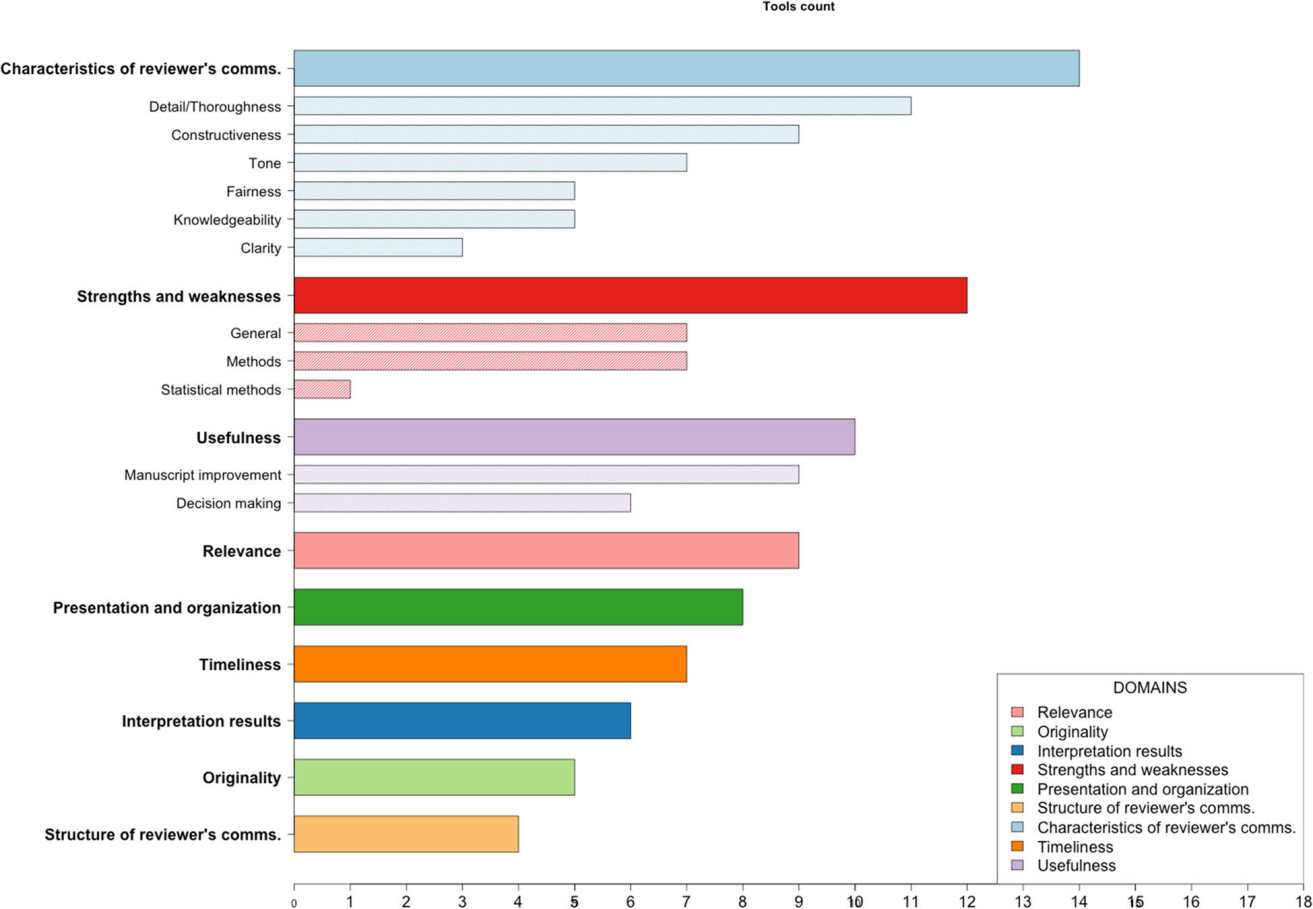
Frequency of quality domains and subdomains

We organized the key concepts from each item into ‘topic-specific matrices’ (Additional file 4), identifying nine main domains and 11 subdomains: 1) relevance of study (*n* = 9); 2) originality of the study (*n* = 5); 3) interpretation of study results (*n* = 6); 4) strengths and weaknesses of the study (*n* = 12) (general, methods and statistical methods); 5) presentation and organization of the manuscript (*n* = 8); 6) structure of the reviewer’s comments (*n* = 4); 7) characteristics of reviewer’s comments (*n* = 14) (clarity, constructiveness, detail/thoroughness, fairness, knowledgeability, tone); 8) timeliness of the review report (*n* = 7); and 9) usefulness of the review report (*n* = 10) (decision making and manuscript improvement). The total number of tools corresponding to each domain and subdomain is shown in Fig. 2. An explanation and example of all domains and subdomains is provided in Table 5. Some domains and subdomains were considered in most tools, such as whether the reviewers’ comments were *detailed/thorough* (*n* = 11) and *constructive* (*n* = 9), whether the reviewers’ comments were on the *relevance of the study* (*n* = 9) and if the peer review report was *useful for manuscript improvement* (*n* = 9). However, other items were rarely considered, such as whether the reviewer made comments on the *statistical methods* (*n* = 1).

**Table 5:** Explanations and Examples of quality domains and subdomains

N	Domains	Subdomains	Explanations and Examples
1	Relevance of the study		Explanation: Items inquiring if the reviewer has discussed in the peer review report the importance of the research question and usefulness of the study. Example: ‘Did the reviewer give appropriate attention to the importance of the question?’ [28]
2	Originality of the study		Explanation: Items inquiring if the reviewer has commented in the peer review report on the originality of the manuscript. Example: ‘Did the reviewer discuss the originality of the paper?’ [23, 27]
3	Interpretation of the study results		Explanation: Items inquiring if the reviewer has commented in the peer review report on how authors interpreted and discussed the results of the study. Example: ‘The reviewer commented accurately and productively on the quality of the author’s interpretation of the data, including acknowledgment of the data’s limitations.’ [26]
4	Strengths and weaknesses of the study	General	Explanation: Items inquiring if the reviewer has identified and commented in the peer review report on the general strong and weak points of the study. Example: ‘How well it identified the study’s strengths and weaknesses?’ [35]
		Methods	Explanation: Items inquiring if the reviewer has identified and commented in the peer review report on the strong and weak points specifically related to study’s methods Example: ‘Did the reviewer clearly identify strengths and weaknesses in the study’s methods?’ [28]
		Statistical methods	Explanation: Items inquiring if the reviewer has identified and commented in the peer review report on the strong and weak points specifically related to study’s statistical methods Example: ‘Confidence intervals/*p*-values/overall fit’ [36]
5	Presentation and organization of the manuscript		Explanation: Items inquiring if the reviewer has made comments in the peer review report on the data presentation such as tables and figures and on the organization of the manuscript such as writing communication. Example: ‘Are there any constructive suggestions on improvement of a. writing; b. data presentation and c. interpretation’ [54]
6	Structure of reviewer’s comments		Explanation: Items inquiring if the reviewer has made in the peer review report organized and structured comments. Example: ‘Concise well-organized comments to the editor’ [50]
7	Characteristics of reviewer’s comments	Clarity	Explanation: Items inquiring if the reviewer has provided in the peer review report clear and easily to read comments. Example: ‘How clear was this review? The review was easily read and interpreted by the editor and authors.’ [38]
		Constructiveness	Explanation: Items inquiring if the reviewer has provided in the peer review report helpful, relevant and realistic comments. Example: ‘Were the reviewer’s comments constructive?’ [23, 27]
		Detail/Thoroughness	Explanation: Items inquiring if the reviewer has provided in the peer review report detailed and thorough comments supplying appropriate evidence. Example: ‘Detail of commentary’ [33]
		Fairness	Explanation: Items inquiring if the reviewer has provided in the peer review report balanced and objective comments. Example: ‘Balanced/fair’ [24, 36]
		Knowledgeability	Explanation: Items inquiring if the reviewer has showed in the peer review report to know and understand correctly the content of the manuscript. Example: ‘Knowledge of the manuscript’s content area.’ [28]
		Tone	Explanation: Items inquiring if the reviewer has used a courteous tone in the peer review report. Example: ‘Overall tone of the reviewers was also assessed as harsh or courteous.’ [34]
8	Timeliness of the review report		Explanation: Items inquiring if the reviewer has completed the peer review report on time. Example: ‘Punctuality of the review’ [49]
9	Usefulness of the review report	Decision making	Explanation: Items inquiring if the reviewer has provided a peer review report useful to make a decision about the acceptance, revision or rejection of a manuscript Example: ‘The reviewer provided the editor with the proper context and perspective to make a decision about acceptance or revision of the manuscript.’ [26]
		Manuscript improvement	Explanation: Items inquiring if the reviewer has provided useful suggestions in the peer review report to improve the manuscript. Example: ‘This aspect is solely interested in how well the review aids the authors for improving their work and/or writing. Whether the review makes a good judgment regarding acceptance of the submission plays no role here whatsoever.’ [53]

### Clustering analysis among tools

**Fig. 3:**
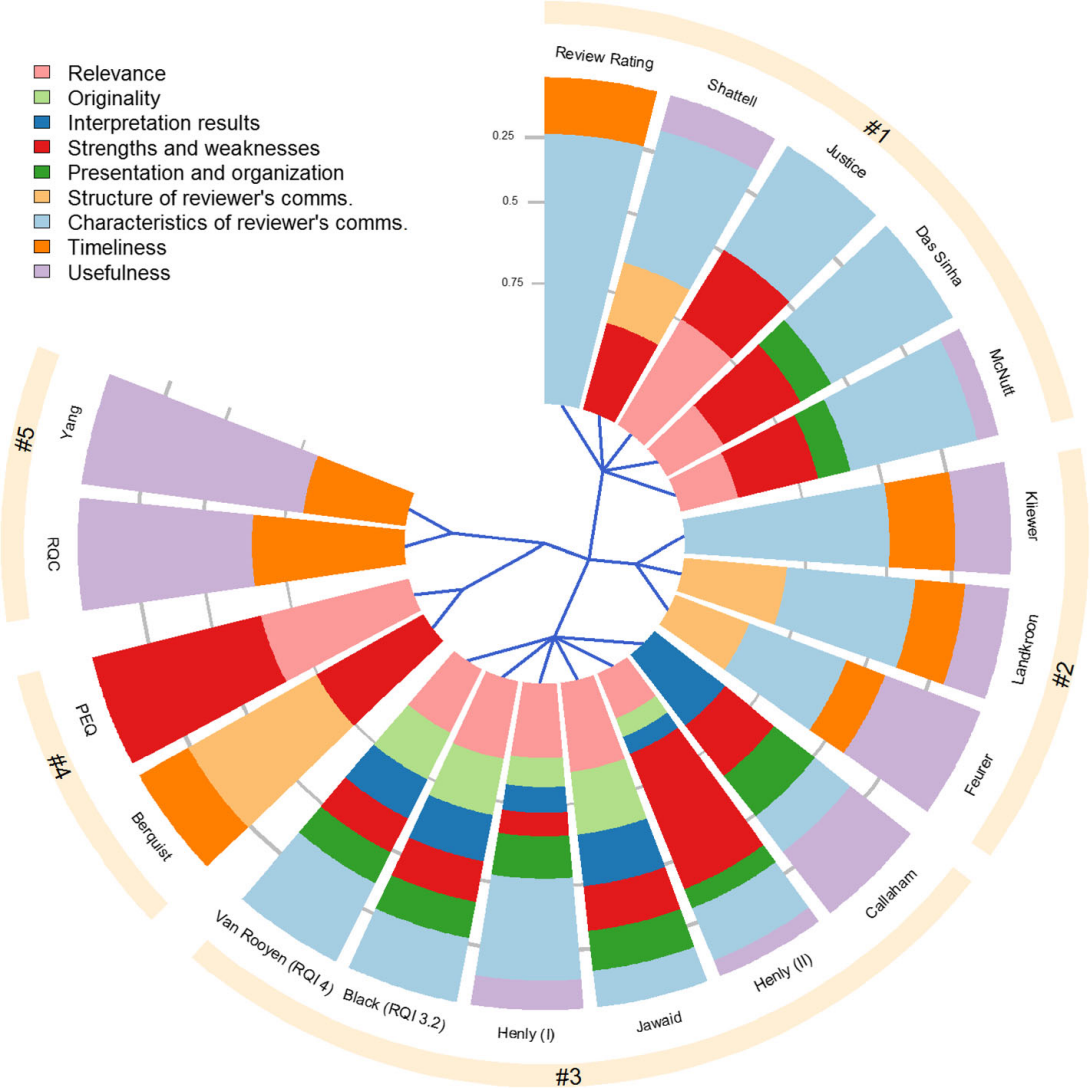
Hierarchical clustering of tools based on the nine quality domains. The figure shows which quality domains are present in each tool. A slice of the chart represents a tool, and each slice is divided into sectors, indicating quality domains (in different colours). The area of each sector corresponds to the proportion of each domain within the tool. For instance, the “Review Rating” tool consists of two domains: *Timeliness*, meaning that 25% of all its items are encompassed in this domain, and *Characteristics of reviewer’s comments* occupying the remaining 75%. The blue lines starting from the centre of the chart define how the tools are divided into the five clusters. Clusters #1, #2 and #3 are sub-nodes of a major node grouping all three, meaning that the tools in these clusters have a similar domain profile compared to the tools in clusters #4 and #5

We created a domain profile for each tool. For example, the tool developed by Justice et al. consisted of 5 items [35]. We classified three items under the domain ‘*Characteristics of the reviewer’s comments*’, one under ‘*Timeliness of the review report*’ and one under ‘*Usefulness of the review report*’. According to the aforementioned classification, the domain profile (represented by proportions of domains) for this tool was 0.6:0.2:0.2 for the incorporating domains and 0 for the remaining ones. The hierarchical clustering used the matrix of Euclidean distances among domain profiles, which led to five main clusters (Fig. 3).

The first cluster consisted of 5 tools developed from 1990 to 2016. All tools included at least one item in the *characteristics of the reviewer’s comments* domain, representing at least 50% of each domain profile. In the second cluster, there were 3 tools developed from 1994 to 2006. These tools were characterized to incorporate at least one item in the *usefulness* and *timeliness* domains. The third cluster included 6 tools that had been developed from 1998 to 2010 and exhibited the most heterogeneous mix of domains. These tools were distinct from the rest because they encompassed items related to *interpretation of the study results* and *originality of the study*. Moreover, the third cluster included two tools with different versions and variations. The first, second, and third cluster were linked together in the hierarchical tree that presented tools with at least one quality component grouped in the domain *characteristics of the reviewer’s comments.* In the fourth cluster, there are 2 tools developed from 2011 to 2017 that consist of at least one component in the *strengths and weaknesses* domain. Finally, the fifth cluster included 2 tools developed from 2009 to 2012 and which consisted of the same 2 domains. The fourth and fifth clusters were separated from the rest in the hierarchical tree that presented tools with only a few domains.

## Discussion

To the best of our knowledge, this is the first comprehensive review that has systematically identified tools used in biomedical research for assessing the quality of peer review reports. We have identified 24 tools from both the medical literature and an internet search: 23 scales and 1 checklist. One out of four tools consisted of a single item that simply asked the evaluator for a direct assessment of the peer review report’s ‘overall quality’. The remaining tools had between 4 to 26 items in which the overall quality was assessed as the sum of all items, their mean, or as a summary score.

Since a definition of overall quality was not provided, these tools consisted exclusively of a subjective quality assessment by the evaluators. Moreover, we found that only one study reported a rigorous development process of the tool, although it included a very limited number of people. This is of concern because it means that the identified tools were, in fact, not suitable to assess the quality of a peer review report, particularly because they lack a focused theoretical basis. We found 10 tools that were evaluated for validity and reliability; in particular, criterion validity was not assessed for any tool.

Most of the scales with more than one item resulted in a summary score. These scales did not consider how items could be weighted differently. Although commonly used, scales are controversial tools in assessing quality primarily because using a score ‘in summarization weights’ would cause a biased estimation of the measured object [43]. It is not clear how weights should be assigned to each item of the scale [18]. Thus different weightings would produce different scales, which could provide varying quality assessments of an individual study [44].

n our methodological systematic review, we found only one checklist. However, it was neither rigorously developed nor validated and therefore we could not consider it adequate for assessing peer review report quality. We believe that checklists may be a more appropriate means for assessing quality because they do not present an overall score, meaning they do not require a weight for the items.

It is necessary to clearly define what the tool measures. For example, the Risk of Bias (RoB) tool [45] has a clear aim (to assess trial conduct and not reporting), and it provides a detailed definition of each domain in the tool, including support for judgment. Furthermore, it was developed with transparent procedures, including wide consultation and review of the empirical evidence. Bias and uncertainty can arise when using tools that are not evidence-based, rigorously developed, validated and reliable; and this is particularly true for tools that are used for evaluating interventions aimed at improving the peer review process in RCTs, thus affecting how trial results are interpreted.

We found that most of the items included in the different tools did not cover the scientific aspects of a peer review report nor were constrained to biomedical research. Surprisingly, few tools included an item related to the methods used in the study, and only one inquired about the statistical methods.

In line with a previous study published in 1990 [28], we believe that the quality components found across all tools could be further organized according to the perspective of either an editor or author, specifically by taking into account the different yet complementary uses of a peer review report. For instance, reviewer’s comments on the *relevance of the study* and *interpretation of the study’s results* could assist editors in making an editorial decision, *clarity* and *detail/thoroughness* of reviewer’s comments are important attributes which help authors improve manuscript quality. We plan to further investigate the perspectives of biomedical editors and authors towards the quality of peer review reports by conducting an international online survey. We will also include patient editors as survey’s participants as their involvement in the peer review process can further ensure that research manuscripts are relevant and appropriate to end-users [46].

The present study has strengths but also some limitations. Although we implemented a comprehensive search strategy for reports by following the guidance for conducting methodological reviews [20], we cannot exclude a possibility that some tools were not identified. Moreover, we limited the eligibility criteria to reports published only in English. Finally, although the number of eligible records we identified through Google® was very limited, it is possible that we introduced selection bias due to a (re)search bubble effect [47].

Due to the lack of a standard definition of quality, a variety of tools exist for assessing the quality of a peer review report. Overall, we were able to establish 9 quality domains. Between two to seven domains were used among each of the 18 tools. The variety of items and item combinations amongst tools raises concern about variations in the quality of publications across biomedical journals. Low-quality biomedical research implies a tremendous waste of resources [48] and explicitly affects patients’ lives. We strongly believe that a validated tool is necessary for providing a clear definition of peer review report quality in order to evaluate interventions aimed at improving the peer review process in well-performed trials.

## Conclusions

The findings from this methodological systematic review show that the tools for assessing the quality of a peer review report have various components, which have been grouped into 9 domains. We plan to survey a sample of editors and authors in order to refine our preliminary classifications. The results from further investigations will allow us to develop a new tool for assessing the quality of peer review reports. This in turn could be used to evaluate interventions aimed at improving the peer review process in RCTs. Furthermore, it would help editors: 1) evaluate the work of reviewers; 2) provide specific feedback to reviewers; and 3) identify reviewers who provide outstanding review reports. Finally, it might be further used to score the quality of peer review reports in developing programs to train new reviewers.

## Additional files

Additional file 1: Search strategies. (PDF 182 kb)

Additional file 2: Excluded studies. (PDF 332 kb)

Additional file 3: Included studies. (PDF 244 kb)

Additional file 4: Classification of peer review report quality components. (PDF 2660 kb)

## Abbreviations

PRISMA: Preferred Reporting Items for Systematic Reviews
RCT: Randomized controlled trials
RoB: Risk of Bias
