# Supplementary material for: Tools used to assess the quality of peer review reports: a methodological systematic review
Source: BMC Med Res Methodol. 2019 Mar 6;19:48. doi: 10.1186/s12874-019-0688-x (PMC6402095; doi:10.1186/s12874-019-0688-x)
Supplement: Supplementary file 1 — Search strategies. (PDF 182 kb) [file 12874_2019_688_MOESM1_ESM.pdf]

Additional file 1. Search strategy

Pubmed (n=2474) (last search: October 27, 2017)

("Peer Review, Research/methods"[Majr] OR "Peer Review, Research/standards"[Majr] OR "Peer Review /methods"[Majr] OR "Peer Review /standards"[Majr]OR peer review*[tiab]) AND (manuscript*[tiab] OR article*[tiab] OR publication*[tiab] OR report[tiab] OR reports[tiab] OR submission*[tiab] OR review*[tiab]) AND ("Quality Control"[Mesh] OR quality[ti] OR validity[ti] OR measure*[ti] OR instrument*[ti] OR scale*[ti] OR score*[ti] OR assessment*[ti] OR apprais*[ti] OR tool[ti])

Cochrane Library (n=340) (last search: October 27, 2017)

ID	Search
#1	MeSH descriptor: [Peer Review, Research] explode all trees and with qualifier(s): [Methods - MT]
#2	MeSH descriptor: [Peer Review, Research] explode all trees and with qualifier(s): [Standards - ST]
#3	MeSH descriptor: [Peer Review] explode all trees and with qualifier(s): [Methods - MT]
#4	MeSH descriptor: [Peer Review] explode all trees and with qualifier(s): [Standards - ST]
#5	"peer review*":ti,ab,kw (Word variations have been searched)
#6	#1 or #2 or #3 or #4 or #5
#7	"manuscript*":ti,ab,kw (Word variations have been searched)
#8	"article*":ti,ab,kw (Word variations have been searched)
#9	"publication*":ti,ab,kw (Word variations have been searched)
#10	"report":ti,ab,kw (Word variations have been searched)
#11	reports:ti,ab,kw (Word variations have been searched)
#12	"submission*":ti,ab,kw (Word variations have been searched)
#13	"review*":ti,ab,kw (Word variations have been searched)
#14	#7 or #8 or #9 or #10 or #11 or #12 or #13
#15	MeSH descriptor: [Quality Control] explode all trees
#16	"quality":ti (Word variations have been searched)
#17	"validity":ti (Word variations have been searched)
#18	"measure*":ti (Word variations have been searched)
#19	"instrument*":ti (Word variations have been searched)
#20	"scale*":ti (Word variations have been searched)
#21	"score*":ti (Word variations have been searched)
#22	"assessment*":ti (Word variations have been searched)
#23	"apprais*":ti (Word variations have been searched)
#24	"tool":ti (Word variations have been searched)
#25	#15 or #16 or #17 or #18 or #19 or #20 or #21 or #22 or #23 or #24
#26	#6 and #14 and #25

EMBASE (n=3698) (last search: October 27, 2017)

('peer review'/exp OR 'peer review*') AND ('methodology'/exp OR 'research'/exp OR 'standards'/exp) AND (manuscript*:ab,ti OR article*:ab,ti OR publication*:ab,ti OR report:ab,ti OR reports:ab,ti OR submission*:ab,ti OR review*:ab,ti) AND ('quality control'/exp OR quality:ti OR validity:ti OR measure*:ti OR instrument*:ti OR scale*:ti OR score*:ti OR assessment*:ti OR apprais*:ti OR tool:ti) AND [embase]/lim

Google® Search (last search: October 20, 2017)

It was conducted using the following terms: peer review, report and quality. The first 200 links were investigated.
