# Supplementary material for: Tools used to assess the quality of peer review reports: a methodological systematic review
Source: BMC Med Res Methodol. 2019 Mar 6;19:48. doi: 10.1186/s12874-019-0688-x (PMC6402095; doi:10.1186/s12874-019-0688-x)
Supplement: Supplementary file 2 — Excluded studies. (PDF 332 kb) [file 12874_2019_688_MOESM2_ESM.pdf]

Additional file 2. Excluded studies and reasons for exclusion (n=34)

References*	Reasons for exclusion
1. Ammenwerth E, Knaup P, Ulmer H, Wolff AC, Haux R. Developing and evaluating criteria to help reviewers of biomedical informatics manuscripts. Informatik Biometrie und Epidemiologie in Medizin und Biologie. 2003;10(5):512-14.	**No tool of interest** (Criteria for reviewers to support an objective high-quality review)
2. Baxt WG, Waeckerle JF, Berlin JA, Callaham ML. Who reviews the reviewers? Feasibility of using a fictitious manuscript to evaluate peer reviewer performance. Annals of emergency medicine. 1998;32(3):310-7.	**No tool of interest** (Number of errors oppositely introduced by the editors)
3. Blank RM. The effects of double-blind versus single-blind reviewing: Experimental evidence from the American Economic Review. The American Economic Review. 1991;81(5)1041-67.	**No outcome of interest** (Paper acceptance rate)
4. Bornmann L, Daniel HD. Do author-suggested reviewers rate submissions more favorably than editor-suggested reviewers? a study on atmospheric chemistry and physics. PLoS ONE. 2010;5(10)1-8.	**No tool of interest** (Assessment of manuscript)
5. Callaham M. Training of peer reviewers: validation of a 5-point rating scale. PLoS medicine. 2007;4:e166.	**Type of reference** (Note of the author to his published manuscript)
6. Cohen IT, Patel K. Peer review interrater concordance of scientific abstracts: A study of anesthesiology subspecialty and component societies. Anesthesia and Analgesia. 2006;102(5):1501-3.	**No tool of interest** (Assessment of abstract)
7. Cummings P. Effects of differences between peer reviewers suggested by authors and by editors. JAMA. 2006;296(10):1231-2.	**No tool involved**
8. Das Sinha S, Sahni P, Nundy S. The effect of informing referees that their comments would be exchanged on the quality of their reviews (abstract) [Internet]. 1997 Available from: https://peerreviewcongress.org/abstracts_1997.html#trev	**Abstract of an included study**
9. EaEarnshaw JJ, Farndon JR, Guillou PJ, Johnson CD, Murie JA, Murray GD. A comparison of reports from referees chosen by authors or journal editors in the peer review process. Annals of the Royal College of Surgeons of England. 2000;82(4 Suppl):133-5.	**No tool of interest** (Assessment of manuscript)
10. Fisher M, Friedman SB, Strauss B. The effects of blinding on acceptance of research papers by peer review. Journal of the American Medical Association. 1994;272(2):143-6.	**No tool of interest** (Assessment of manuscript)
11. Godlee F, Gale CR, Martyn CN. Effect on the quality of peer review of blinding reviewers and asking them to sign their reports: a randomized controlled trial. JAMA. 1998;280(3):237-40.	**No tool of interest** (Number of weaknesses oppositely introduced by the editors)
12. Godlee F, Gale CR, Martyn CN. The effect on the quality of peer review of blinding reviewers and asking them to sign their reports: a randomized controlled trial [abstract] [Internet]. 1997 Available from: https://peerreviewcongress.org/abstracts_1997.html#thtr	**No tool of interest** (Number of weaknesses oppositely introduced by the editors)
13. Green SM, Callaham ML. Implementation of a journal peer reviewer stratification system based on quality and reliability. Annals of emergency medicine. 2011;57(2):149-52.	**No tool of interest** (Peer Reviewer Stratification System)
14. Groves T. Best practice in peer review and editing,	**No tool involved**

ensuring article quality. Notfall und Rettungsmedizin. 2010;13(1):6-8.	
15. Helton M, Balistreri W. Assessment of reviewers recommended by authors vs editors: is there bias? (abstract) [Internet]. 2009 Available from: https://peerreviewcongress.org/abstracts_2009.html#81	**No outcome of interest**
16. Hwang K, Hwang SH. Is Double-Blinded Peer Review Necessary? The Effect of Blinding on Review Quality. Plastic and reconstructive surgery. 2016;138(1):161e-2e.	**No tool involved**
17. Jadad AR, Moore RA, Carroll D, Jenkinson C, Reynolds DJ, Gavaghan DJ, McQuay HJ. Assessing the quality of reports of randomized clinical trials: is blinding necessary?. Controlled clinical trials. 1996;17(1):1-2.	**No tool of interest**<br>(Assessment of RCTs report)
18. Janke KK, Bzowyckyj AS, Traynor AP. Editors' perspectives on enhancing manuscript quality and editorial decisions through peer review and reviewer development. American Journal of Pharmaceutical Education. 2017;81(4):73.	**No outcome of interest**<br>(Manuscript quality)
19. Jurkat-Rott K, Lehmann-Horn F. Reviewing in science requires quality criteria and professional reviewers. European journal of cell biology. 2004;83(3):93-5.	**No tool involved**
20. Lee SS. How to be a great reviewer: an editor's view. Liver International. 2008;28(2):158-9.	**No tool involved**
21. Marchionini G. Rating reviewers. Science. 2008;319(5868):1335-6.	**No tool involved**
22. McNutt R, Glass RM. Peer reviewer recommendations and ratings of manuscript quality for accepted and rejected manuscripts (abstract) [Internet]. 2001. Available from: https://peerreviewcongress.org/abstracts_2001.html#rejected	**No tool involved**
23. Moore A. What's in a peer review report?. Bioessays. 2013;35(2):77-.	**No tool involved**
24. Okike K, Hug KT, Kocher MS, Leopold SS. Single-blind vs double-blind peer review in the setting of author prestige. JAMA. 2016;316(12):1315-6.	**No tool of interest**<br>(Number of errors oppositely introduced by the editors)
25. Open peer review is feasible and does not reduce quality of reviews. BMJ. 1999;318:d.	**Type of reference**<br>(Part of the introductive page "This week in the BMJ")
26. Parikh L, Benner RS, Riggs TW, Chescheir NC. Factors influencing review quality and reviewer recommendation for a high-impact ob-gyn journal. Obstetrics and Gynecology. 2016;127:139S.	**No tool involved**
27. Polak JF. The role of the manuscript reviewer in the peer review process. AJR. American journal of roentgenology. 1995;165(3):685-8.	**No tool of interest**<br>(Monitor reviewer's performance)
28. Resnik DB, Elmore SA. Ensuring the Quality, Fairness, and Integrity of Journal Peer Review: A Possible Role of Editors. Science and engineering ethics. 2016;22(1):169-88.	**No tool involved**
29. Richards D. Little evidence to support the use of editorial peer review to ensure quality of published research. Evidence-based dentistry. 2007;8(3):88-9.	**No outcome of interest**<br>(Manuscript quality)
30. Rogers LF. Peer reviewers: reviewing manuscripts for the AJR. (editorial) AJR 2002;178(5):1051–1052	**No tool of interest**<br>(Assessment of manuscript)
31. Shauver MJ, Chung KC. Reply: Is Double-Blinded Peer Review Necessary? The Effect of Blinding on Review Quality. Plastic and reconstructive surgery. 2016;138(1):162e-3e.	**No tool involved**
32. Silobrčić V. Relative scales and their possible use in	**No tool of interest**

evaluation of scientific research in a small scientific community. Acta Medica Croatica. 2004;58(3):173-6.	(Assessment of manuscript)
33. Szekely T, Kruger O, Krause ET. Errors in science: the role of reviewers. Trends in ecology & evolution. 2014;29(7):371-3.	**No tool involved**
34. Tonks A. Reviewers chosen by authors. May be better than reviewers chosen by editors. British Medical Journal. 1995;311(6999):210.	**No tool of interest**<br>(Evaluation of journal´s review process)

*In alphabetical order
