# Supplementary material for: Tools used to assess the quality of peer review reports: a methodological systematic review
Source: BMC Med Res Methodol. 2019 Mar 6;19:48. doi: 10.1186/s12874-019-0688-x (PMC6402095; doi:10.1186/s12874-019-0688-x)
Supplement: Supplementary file 3 — Included studies. (PDF 244 kb) [file 12874_2019_688_MOESM3_ESM.pdf]

Additional file 3. Included studies (n=46)

1. Almquist M, Von Allmen RS, Carradice D, Oosterling SJ, McFarlane K, Wijnhoven B. A prospective study on an innovative online forum for peer reviewing of surgical science. PLoS One. 2017;12(6):1–13.
2. Berquist T. Improving your reviewer score: It’s not that difficult. AJR. 2017;209:711–2.
3. Bingham CM, Higgins G, Coleman R, Van Der Weyden MB. The Medical Journal of Australia internet peer-review study. The Lancet. 1998;352(9126):441-5.
4. Black N, Van Rooyen S, Godlee F, Smith R, Evans S. What makes a good reviewer and a good review for a general medical journal?. JAMA. 1998;280(3):231-3.
5. Callaham ML, Baxt WG, Waeckerle JF, Wears RL. Reliability of editors' subjective quality ratings of peer reviews of manuscripts. JAMA. 1998;280(3):229-31.
6. Callaham ML, Knopp R, John Gallagher E. Effect of written feedback by editors on quality of reviews: Two randomized trials. JAMA. 2002;287(21):2781–3.
7. Callaham ML, McCulloch C. Longitudinal trends in the performance of scientific peer reviewers. Ann Emerg Med. 2011;57(2):141-8.
8. Callaham ML, Schriger DL. Effect of Structured Workshop Training on Subsequent Performance of Journal Peer Reviewers. Ann Emerg Med. 2002;40(3):323–8.
9. Callaham ML, Tercier J. The Relationship of Previous Training and Experience of Journal Peer Reviewers to Subsequent Review Quality. 2007;4(1):0032–40.
10. Callaham ML, Wears RL, Waeckerle JF. Effect of attendance at a training session on peer reviewer quality and performance. Ann Emerg Med.1998;32(3):318-22.
11. Chung KC, Shauver MJ, Malay S, Zhong L, Weinstein A, Rohrich RJ. Is Double-Blinded Peer Review Necessary? the Effect of Blinding on Review Quality. Plast Reconstr Surg. 2015;136(6):1369–77.
12. Das Sinha S., Sahni P, Nundy S. Does exchanging comments of Indian and non-Indian reviewers improve the quality of manuscript reviews ? Natl Med J India. 1999;12(5):210–3.
13. Evans AT, McNutt RA, Fletcher SW, Fletcher RH. The characteristics of peer reviewers who produce good-quality reviews. J Gen Intern Med. 1993;8(8):422-8.
14. Feurer ID, Becker GJ, Picus D, Ramirez E, Darcy MD, Hicks ME. Evaluating peer reviews. JAMA. 1994;272(2):98-100.
15. Friedman DP. Manuscript peer review at the AJR: facts, figures, and quality assessment. AJR. 1995;164(4):1007-9.
16. Hettyey A, Griggio M, Mann M, Raveh S, Schaedelin FC, Thonhauser KE, et al. Peerage of Science: Will it work? Trends Ecol Evol. 2012;27(4):189–90.
17. Henly SJ, Bennett JA, Dougherty MC. Scientific and statistical reviews of manuscripts submitted to Nursing Research: Comparison of completeness, quality, and usefulness. Nurs Outlook. 2010;58(4):188-99.
18. Henly SJ, Dougherty MC. Quality of manuscript reviews in nursing research. Nurs Outlook. 2009 Jan 1;57(1):18-26.
19. Houry D, Green S, Callaham M. Does mentoring new peer reviewers improve review quality ? A randomized trial. BMC Med Educ. 2012;12(83):1–7
20. Jawaid SA, Jawaid M, Jafary MH. Characteristics of reviewers and quality of reviews: a retrospective study of reviewers at Pakistan Journal of Medical Sciences. Pak J Med Sci. 2006;22(2):101-6.

21. Justice AC, Cho MK, Winker MA, Berlin JA, Rennie D, Peer Investigators. Does masking author identity improve peer review quality?: A randomized controlled trial. JAMA. 1998;280(3):240-2.
22. Kliewer MA, Freed KS, DeLong DM, Pickhardt PJ, Provenzale JM. Reviewing the reviewers: comparison of review quality and reviewer characteristics at the American Journal of Roentgenology. AJR. 2005;184(6):1731-5.
23. Kowalczuk MK, Dudbridge F, Nanda S, Harriman SL, Patel J, Moylan EC. Retrospective analysis of the quality of reports by author-suggested and non-author-suggested reviewers in journals operating on open or single-blind peer review models. BMJ Open. 2015;5(e008707):1–10
24. Landkroon AP, Euser AM, Veeken H, Hart W, Overbeke AJ. Quality assessment of reviewers' reports using a simple instrument. Obstet Gynecol. 2006;108(4):979-85.
25. Moore A, Jones R. Supporting and enhancing peer review in the BJGP. Br J Gen Pract. 2014;64(624):e459-61.
26. McNutt RA, Evans AT, Fletcher RH, Fletcher SW. The effects of blinding on the quality of peer review: a randomized trial. JAMA. 1990;263(10):1371-6.
27. Rajesh A, Cloud G, Harisinghani MG. Improving the quality of manuscript reviews: Impact of introducing a structured electronic template to submit reviews. AJR. 2013;200(1):20-3.
28. Pitkin RM, Burmeister LF. Identifying manuscript reviewers: randomized comparison of asking first or just sending. JAMA. 2002;287(21):2795-6.
29. Prechelt L. Review Quality Collector [Internet]. Available from: https://reviewqualitycollector.org/static/pdf/rqdef-example.pdf
30. Publons. Publons for Editors : Overview [Internet]. Available from: https://static1.squarespace.com/static/576fcda2e4fcb5ab5152b4d8/t/58e21609d482e9ebf98163be/14912117
31. Rivara FP, Cummings P, Ringold S, Bergman AB, Joffe A, Christakis DA. A Comparison of Reviewers Selected by Editors and Reviewers Suggested by Authors. J Pediatr. 2007;151(2):202–5.
32. Schroter S, Black N, Evans S, Carpenter J, Godlee F, Smith R. Effects of training on quality of peer review: randomised controlled trial. BMJ. 2004;328(7441):673.
33. Schroter S, Tite L, Hutchings A, Black N. Differences in review quality and recommendations for publication between peer reviewers suggested by authors or by editors. JAMA. 2006;295(3):314-7
34. Schroter S, Black N, Evans S, Godlee F, Osorio L, Smith R. What errors do peer reviewers detect, and does training improve their ability to detect them? J R Soc Med. 2008;101(10):507–14.
35. Shattell MM, Chinn P, Thomas SP, Cowling WR. Authors ' and Editors ' Perspectives on Peer Review Quality in Three Scholarly Nursing Journals. J Nurs Scholarsh. 2010;42(1):58–65.
36. Stossel TP. Reviewer status and review quality. N Engl J Med. 1985;312(10):658–9.
37. Thompson SR, Agel J, Losina E. The JBJS Peer-Review Scoring Scale : A valid, reliable instrument for measuring the quality of peer review reports. Learn Publ. 2016;29:23–5.
38. Van Rooyen S, Black N, Godlee F. Development of the review quality instrument (RQI) for assessing peer reviews of manuscripts. J Clin Epidemiol. 1999;52(7):625-9.
39. Van Rooyen S, Delamothe T, Evans SJ. Effect on peer review of telling reviewers that their signed reviews might be posted on the web: randomised controlled trial. BMJ. 2010;341:c5729.
40. Van Rooyen S, Godlee F, Evans S, Black N, Smith R. Effect of open peer review on quality of reviews and on reviewers' recommendations: a randomised trial. BMJ. 1999;318(7175):23-7.

41. Van Rooyen S, Godlee F, Evans S, Smith R, Black N. Effect of Blinding and Unmasking on the Quality of Peer Review. JAMA. 1998;280(3):234–7.
42. Vinther S, Nielsen OH, Rosenberg J, Keiding N, Schroeder T V. Same review quality in open versus blinded peer review in ‘ Ugeskrift for Læger ’. Dan Med J. 2012;59(8):1–5.
43. Wager E, Parkin EC, Tamber PS. Are reviewers suggested by authors as good as those chosen by editors? Results of a rater-blinded, retrospective study. BMC Med. 2006;32(3):61–4.
44. Walsh E, Rooney M, Appleby L, Wilkinson G. Open peer review : a randomised controlled trial. Br J Psychiatry. 2000;176:47–51.
45. Weber EJ, Katz PP, Waeckerle JF, Callaham ML. Author perception of peer review: impact of review quality and acceptance on satisfaction. JAMA. 2002;287(21):2790-3.
46. Yang Y. Effects of Training Reviewers on Quality of Peer Review: A Before-and-After Study (Abstract) [Internet]. 2009. Available from: https://peerreviewcongress.org/abstracts_2009.html
