# Supplementary material for: Tools used to assess the quality of peer review reports: a methodological systematic review
Source: BMC Med Res Methodol. 2019 Mar 6;19:48. doi: 10.1186/s12874-019-0688-x (PMC6402095; doi:10.1186/s12874-019-0688-x)
Supplement: Supplementary file 4 — Classification of peer review report quality components. (PDF 2660 kb) [file 12874_2019_688_MOESM4_ESM.pdf]

Appendix 4. Classification of peer review report quality components

Peer Review Report Quality Components	N. of tools
**1. Relevance of the study**<br>• Did the reviewer discuss the importance of the research question? (1,2)<br>• Importance of question: Did the reviewer pay appropriate attention to the importance of the research question? (3)<br>• Did the reviewer give appropriate attention to the importance of the question? (4)<br>• Usefulness of the study (5)<br>• How well the review addressed the clinical or research importance of the study? (6)<br>• Discussion: measures the merit of identifying and discussing the importance, implications or improvements of the research (7)<br>• Theoretical framework (8,9)<br>• Literature review/ references (8,9)	9
**2. Originality of the study**<br>• Did the reviewer discuss the originality of the paper? (1,2)<br>• Problem statement/originality (8,9)<br>• Originality of manuscripts (5)	5
**3. Interpretation of the results**<br>• Did the reviewer comment on the author´s interpretation of the results? (1,2)<br>• The reviewer commented accurately and productively on the quality of the author´s interpretation of the data, including acknowledgment of the data's limitations (10)<br>• Discussion/ Interpretation of results (8,9)<br>• Interpretation of results (5)	6
**4. Strengths and weaknesses of the study**	
4.1 General<br>• Comments enhance the merits and relevance of the work (11)<br>• Strong and weak points (5)<br>• How well it identified the study´s strengths and weaknesses? (6)<br>• Merits: measures how accurate and justified the review is in identifying manuscript strengths (7)<br>• Critique: measures how accurate the and justified the review is in identifying manuscript weaknesses (7)<br>• Shortcomings identified (12)<br>• Target key issues: Did the reviewer concisely identify the key issues that either make or break the manuscript (from the editor's perspective)? (3)<br>• Did the reviewer target key issues? (4)	7

4.2 Methods<br>• Did the reviewer clearly identify the strengths and weaknesses of the methods (study design, data collection and data analysis)? (1,2)<br>• The reviewer identified and commented on major strengths and weaknesses of study design and methods (10)<br>• Methods: Did the reviewers clearly identify the strengths and weaknesses of the a. study design; b. data collection and c. data analysis (3)<br>• Did the reviewer clearly identify strengths and weaknesses in the study´s methods? (4)<br>• Design/methods (8,9)	7
4.3 Statistical methods<br>• Persons (9)<br>• Occasions (9)<br>• Variables and measurement (9)<br>• Procedure (9)<br>• Data quality (9)<br>• Model/estimator/assumptions (9)<br>• Confidence intervals/p-values/overall fit (9)<br>• Statistical interpretation (9)<br>• Tables (9)<br>• Graphs (9)	1
**5. Presentation and organization of the manuscript**<br>• Did the reviewer make specific useful comments on the writing, organisation, tables and figures of the manuscript? (1,2)<br>• The reviewer commented on mayor strengths and weaknesses of the manuscript as a written communication, independent of the design, methods, results, and interpretation of the study (10)<br>• Presentation: Are there any constructive suggestions on improvement of a. writing; b. data presentation and c. interpretation (3)<br>• Did the reviewer make constructive comments about the quality of writing and presentation of data? (4)<br>• Data analysis/Presentation (8,9)<br>• Organization/writing (8,9)<br>• Tables and figures (5)	8
**6. Structure of reviewer´s comments**<br>• Sophisticated detailed comments to the author by section with line and page references (11)<br>• Concise well-organized comments to the editor (11)<br>• Section-by-section review (13)<br>• Structure (14)<br>• Consistent with journal's review criteria (12)	4
**7. Characteristics of reviewers' comments**<br>7.1 Clarity<br>• Clear (8,9)<br>• How clear was this review? The review was easily read and interpreted by the editor and authors (17)	3

7.2 Constructiveness<br>• Were the reviewer´s comments constructive? (1,2)<br>• The reviewer´s comments to author were constructive and professional (10)<br>• Constructive (3,8,9)<br>• Constructiveness (4,14)<br>• How helpful was this review? Comments were constructive, relevant, and realistic (17)	9
7.3 Detail/ Thoroughness<br>• The amount of detail (16)<br>• Level of sophistication of the commentary (16)<br>• Detail of commentary (12)<br>• General: Was the reviewer a. thorough (3)<br>• Thoroughness (4)<br>• Precise (8,9)<br>• How thorough was this review? The review gave adequate consideration to all aspects of the paper including methodology, figures, interpretation and presentation of results, ethics, relevance, etc. (17)<br>• Did the reviewer supply appropriate evidence using examples from the paper to substantiate their comments? (1,2)<br>• Offering supporting references (13)<br>• Did reviewers supply evidence to support their statements? (6)<br>• Logical (8,9)	11
7.4 Fairness<br>• Fair (3)<br>• Fairness (4)<br>• Balanced/fair (8,9)<br>• Objectivity (14)	5
7.5 Knowledgeable<br>• Knowledgeable (3)<br>• Knowledge of the manuscript´s content area (4)<br>• Knowledgeable/substantiated (8,9)<br>• Understands content (12)	5
7.6 Tone<br>• How would you rate the tone of the review? (2)<br>• Etiquette (13)<br>• Courteous (3)<br>• Courteousness (4)<br>• Overall tone of the reviewers was also assessed as harsh or courteous (5)<br>• Were reviewers courteous? (6)<br>• Constructive tone (12)	7
**8. Timeliness of the review report**<br>• Timely (14 days) or early review completion (11)<br>• Timeliness (13)<br>• Aspect: Timeliness (15)<br>• Punctuality of the review (16)<br>• Turnaround time (14)<br>• How timely was this review? The review assignment was completed within the time limits established by the editor (17)<br>• Time taken to review (<4 weeks) (18)	7
**9. Usefulness of the review report**<br>9.1 Decision making<br>• Grade sheet (13)	6

• Summary and/or recommendation (13)<br>• The reviewer provided the editor with the proper context and perspective to make a decision about acceptance or revision of the manuscript (10)<br>• Summary grade (4)<br>• Aspect: Helpfulness for Decision (weight 27): This aspect should be evaluated regardless of how useful the review will be as feedback to the authors (15)<br>• Usefulness to editor (8,9)	
9.2 Manuscript improvement<br>• Aspect: Helpfulness for Authors (weight 19): This aspect is solely interested in how well the review aids the authors for improving their work and/or writing. Whether the review makes a good judgment regarding acceptance of the submission plays no role here whatsoever (15)<br>• Perceived Usefulness to authors (8,9)<br>• ≥ 300 words or more than 4 suggestions for improvement (18)<br>• Suggestions to correct errors (18)<br>• Specific errors identified (18)<br>• Better references (18)<br>• The reviewer provided the author with useful suggestions for improvement of the manuscript (10)<br>• The quality of the suggestions for manuscript improvement (16)<br>• Specific suggestions (12)<br>• Insight (14)<br>• New insights/perspectives (13)	9

References

1. Black N, Van Rooyen S, Godlee F, Smith R, Evans S. What makes a good reviewer and a good review for a general medical journal? JAMA. 1998;280(3):231–3.

2. Van Rooyen S, Godlee F, Evans S, Black N, Smith R. Effect of open peer review on quality of reviews and on reviewers ' recommendations: a randomised trial. BMJ. 1999;318(7175):23–7.

3. Das Sinha S., Sahni P, Nundy S. Does exchanging comments of Indian and non-Indian reviewers improve the quality of manuscript reviews? Natl Med J India. 1999;12(5):210–3.

4. Mcnutt RA, Evans AT, Fletcher RH, Fletcher SW. The Effects of Blinding on the Quality of Peer Review. JAMA. 1990;263(10):1371–6.

5. Jawaid SA, Jawaid M, Jafary MH. Characteristics of reviewers and quality of reviews: A retrospective study of reviewers at Pakistan Journal of Medical Sciences. Pakistan J Med Sci. 2006;22(2):101–6.

6. Justice AC, Cho MK, Winker MA, Berlin JA. Does Masking Author Identity Improve Peer Review Quality? A Randomized Controlled Trial. JAMA. 1998;280(3):240–2.

7. Hettyey A, Griggio M, Mann M, Raveh S, Schaedelin FC, Thonhauser KE, et al. Peerage of Science: Will it work? Trends Ecol Evol. 2012;27(4):189–90.

8. Henly SJ, Dougherty MC. Quality of manuscript reviews in nursing research. Nurs Outlook. 2009;57(1):18–26.

9. Henly SJ, Bennett JA, Dougherty MC. Scientific and statistical reviews of manuscripts submitted to Nursing Research: Comparison of completeness, quality, and usefulness. Nurs Outlook. 2010;58(4):188–99.

10. Callaham M, Knopp R, John Gallagher E. Effect of written feedback by editors on quality of reviews: Two randomized trials. JAMA. 2002;287(21):2781–3.

11. Berquist T. Improving your reviewer score: It's not that difficult. AJR. 2017;209(4):711–2.

12. Shattell MM, Chinn P, Thomas SP, Cowling WR. Authors ' and Editors ' Perspectives on Peer Review Quality in Three Scholarly Nursing Journals. J Nurs Scholarsh. 2010;42(1):58–65.

13. Feurer I, Becker G, Picus D, Ramirez E, Darcy M, Hicks M. Evaluating peer reviews: Pilot testing of a grading instrument. JAMA. 1994;272(2):98–100.

14. Landkroon AP, Euser AM, Veeken H. Quality Assessment of Reviewers ' Reports Using a Simple Instrument. Obstet Gynecol. 2006;108(4):979–85.

15. Prechelt L. Review Quality Collector [Internet]. Available from: https://reviewqualitycollector.org/static/pdf/rqdef-example.pdf

16. Kliewer MA, Freed KS, DeLong DM, Pickhardt PJ, Provenzale JM. Reviewing the reviewers: Comparison of review quality and reviewer characteristics at the American Journal of Roentgenology. AJR. 2005;184(6):1731–5.

17. Publons. Publons for Editors: Overview [Internet]. Available from: https://static1.squarespace.com/static/576fcda2e4fcb5ab5152b4d8/t/58e21609d482e9ebf98163be/14912117

18. Yang Y. Effects of Training Reviewers on Quality of Peer Review: A Before-and-After Study (Abstract) [Internet]. 2009. Available from: https://peerreviewcongress.org/abstracts_2009.html
